# No G-Quadruplex Structures in the DNA of Parvovirus B19: Experimental Evidence versus Bioinformatic Predictions

**DOI:** 10.3390/v12090935

**Published:** 2020-08-25

**Authors:** Gloria Bua, Daniele Tedesco, Ilaria Conti, Alessandro Reggiani, Manuela Bartolini, Giorgio Gallinella

**Affiliations:** Department of Pharmacy and Biotechnology, University of Bologna, 40126 Bologna, Italy; gloria.bua2@unibo.it (G.B.); daniele.tedesco@isof.cnr.it (D.T.); ilaria.conti@unife.it (I.C.); alessandro.reggiani5@unibo.it (A.R.); manuela.bartolini3@unibo.it (M.B.)

**Keywords:** parvovirus B19, G-quadruplex, bioinformatics, antivirals, BRACO-19, pyridostatin

## Abstract

Parvovirus B19 (B19V), an ssDNA virus in the family Parvoviridae, is a human pathogenic virus, responsible for a wide range of clinical manifestations, still in need of effective and specific antivirals. DNA structures, including G-quadruplex (G4), have been recognised as relevant functional features in viral genomes, and small-molecule ligands binding to these structures are promising antiviral compounds. Bioinformatic tools predict the presence of potential G4 forming sequences (PQSs) in the genome of B19V, raising interest as targets for antiviral strategies. Predictions locate PQSs in the genomic terminal regions, in proximity to replicative origins. The actual propensity of these PQSs to form G4 structures was investigated by circular dichroism spectroscopic analysis on synthetic oligonucleotides of corresponding sequences. No signature of G4 structures was detected, and the interaction with the G4 ligand BRACO-19 (*N*,*N*′-(9-{[4-(dimethylamino)phenyl]amino}acridine-3,6-diyl)bis(3-pyrrolidin-1-ylpropanamide) did not appear consistent with the stabilisation of G4 structures. Any potential role of PQSs in the viral lifecycle was then assessed in an in vitro infection model system, by evaluating any variation in replication or expression of B19V in the presence of the G4 ligands BRACO-19 and pyridostatin. Neither showed a significant inhibitory activity on B19V replication or expression. Experimental challenge did not support bioinformatic predictions. The terminal regions of B19V are characterised by relevant sequence and symmetry constraints, which are functional to viral replication. Our experiments suggest that these impose a stringent requirement prevailing over the propensity of forming actual G4 structures.

## 1. Introduction

Parvovirus B19 (B19V), an ssDNA virus in the family Parvoviridae [1], is a human pathogenic virus, widely circulating in the population, responsible for an ample spectrum of clinical manifestations [2]. The genome is a 5.6 kb ssDNA molecule of either polarity, with a coding repertoire comprising a non-structural (NS) protein, functional to virus replication, and two structural proteins, VP1 and VP2, constituting a T = 1, 22 nm icosahedral capsid [3,4]. The virus is characterised by a selective but not exclusive tropism for erythroid progenitor cells (EPCs) in the bone marrow and by a strict dependence on the cellular machinery and environment for its replication [5,6].

The selective tropism of B19V for EPCs in the bone marrow and the ability to induce cell cycle arrest and apoptosis in productively infected cells can cause a partial block in erythropoiesis. This may manifest as a transient or persistent erythroid aplasia, clinically acute and severe in patients with underlying haematological disorders, or chronic in patients with immune system deficits [7]. The virus is capable of infecting and maintaining long-term persistence in disparate tissues, mostly within endothelial or stromal cells, and can establish a complex relationship with the immune system, whose efficacy in innate and adaptive responses is crucial to the course of infection and the development of pathological processes [8,9]. In addition to haematological consequences, B19V infection can commonly manifest as erythema infectiosum and cause post-infection arthropathies. Further, a wide range of other different pathologies have been reported, among them mainly myocarditis [10] and autoimmune processes [11]. Infection in pregnancy may be transmitted to the foetus, posing a risk of foetal death and/or foetal hydrops [12,13,14].

B19V infection requires diagnostic awareness to lead and support clinical care in severe cases [15]. The development of antiviral strategies directed against B19V as compared to other viruses is still lagging, although recent work has identified a few compounds that show a selective inhibition of B19V replication in vitro [16]. Such compounds include hydroxyurea (HU), a ribonucleotide reductase inhibitor, also used for the treatment of sickle-cell disease and known to have “virostatic” properties [17]; the nucleotide analogues cidofovir (CDV) and its lipid derivative brincidofovir (BCV), broad-spectrum anti-viral agents mostly active against dsDNA viruses [18,19,20]; and a few coumarin derivatives [21]. Some flavonoid compounds can inhibit the endonuclease activity of viral NS protein, a function critical to the replicative process of B19V [22].

The genome of B19V has a limited coding potential, and its replication depends largely on the cellular environment. Consequently, a deeper understanding of the viral lifecycle and virus–cell interactions are required to identify further targets and agents for an effective antiviral strategy. Unconventional DNA structures have been recognised as relevant features for the regulation of several biological processes, including replication, recombination, and transcription [23]. Particular emphasis has been given to the potential of G-rich sequences to adopt G-quadruplex (G4) planar structures disrupting the regular double-helix structure of DNA [24]. These structures are characterised by stacks of guanine tetrads, which are bound via Hoogsteen-type hydrogen bonds, and can typically form when runs of 2–4 guanine bases are regularly spaced on the DNA sequence. Small-molecule ligands recognising and binding to these structures, either with interfering or with stabilising effects, may act as modulators in the biological process involved, raising interest as compounds of pharmacological interest [25].

Methodological developments have allowed the in silico prediction of specific G4 structures directly from primary sequences, and the number of studies reporting genome-wide G4 exploration across species has rapidly increased [26], including viruses [27]. A recent survey of viral genomes by a regular expression patterns search has led to assembly of a comprehensive database (G4-virus) reporting the presence, distribution, and statistical significance of potential quadruplex sequences (PQSs) in reference genomes and genome sets for all viral families [28]. In some cases, the presence and a biological role of PQS structures in viral genomes have been validated in experimental models, and the role as antiviral agents of specific G4-ligands such as pyridostatin (PDS) and BRACO-19 (*N*,*N*′-(9-{[4-(dimethylamino)phenyl]amino}acridine-3,6-diyl)bis(3-pyrrolidin-1-ylpropanamide) demonstrated in relevant instances [29]. Within the G4-virus database, indication for the presence of PQSs in the B19V genome was reported, raising the need for an experimental challenge of the bioinformatic prediction and, as a consequence, for the investigation of any possible relevance of these structures as targets for antivirals against B19V.

On these grounds, we carried out a closer bioinformatic inspection of the B19V genome for the presence of PQSs, comparing the results reported in the G4-virus database to targeted predictions obtained by a different computational method for G-quadruplex prediction, the QGRS (Quadruplex forming G-Rich Sequences) mapper [30], a method based on a scoring algorithm. By this analysis, we identified two sequence stretches located in the genomic terminal regions, close to the origins of replication of viral DNA, as a potentially relevant PQSs. Experiments were carried out to test the prediction. Synthetic oligonucleotides corresponding to the PQSs were investigated by circular dichroism (CD) spectroscopy, which can provide information on the propensity to form G4 structures. Then, any potential role of PQSs in the viral lifecycle was assessed by using G4 ligands in a model virus–cell system and evaluating the occurrence of a dose-dependent variation in replication or expression levels of B19V.

## 2. Materials and Methods

### 2.1. Bioinformatic Analysis

The B19V sequence used in bioinformatic analysis is a derived consensus sequence, referred to as B19V EC [GenBank KY940273] [31]. The G4-virus PQS database [28] was accessed at http://www.medcomp.medicina.unipd.it/main_site/doku.php?id=g4virus. The QGRS Mapper web server [30] was accessed at http://bioinformatics.ramapo.edu/QGRS/index.php.

### 2.2. Chemicals

Oligonucleotides used in CD analysis (Table 1) were obtained from Eurofins Genomics (Ebersberg, Germany) (https://www.eurofinsgenomics.eu/). BRACO-19 and pyridostatin were obtained from Merck-Sigma (Milan, Italy). Stock solutions were prepared in H_2_O at 1 mM and further diluted for subsequent experiments.

### 2.3. CD Analysis

Circular dichroism (CD) studies on oligonucleotides were carried out on a Jasco (Tokyo, Japan) J-810 spectropolarimeter equipped with a PTC-423S Peltier-type temperature control system. Measurements were performed using a micro-volume QS quartz cell with black walls (1 cm path length, 500 μL volume; Hellma Italia, Milan, Italy). Oligonucleotides and BRACO-19 were diluted from stock solution into an analysis buffer (KCl 70 mM, potassium acetate 20 mM, pH 6.8) at 2 and 10 μM, respectively. PDS was not used in CD studies because its addition to oligonucleotides caused precipitation in the samples, making it unsuitable for spectroscopic analysis. CD spectra (330–230 nm) were recorded at 17 different temperatures (every 5 °C between 15 and 95 °C) applying a 0.25 °C/min gradient for both heating and cooling ramps. A 4 nm spectral bandwidth, a 0.2 nm data interval, a 100 nm/min scanning speed and a 2 s data integration time were employed for measurements; solvent-corrected spectra were then converted to molar units per residue ($\Delta \varepsilon_{\mathrm{res}}$, in M^−1^ cm^−1^). CD melting curves were determined by plotting the $\Delta \varepsilon_{\mathrm{res}}$ values as a function of temperature (T) for each oligonucleotide, using the wavelength at which the difference between their CD signals at 15 and 95 °C was maximum ($\lambda_{\Delta_{\max}}$). Mid-transition temperatures ($T_{m}$) for both heating and cooling ramps were then derived by non-linear regression on the CD melting curves using a 6-parameter logistic function [32,33].

### 2.4. Cells

Erythroid progenitor cells (EPCs) were generated in vitro from peripheral blood mononuclear cells (PBMC), as described [5]. Blood donations were made available for institutional research purposes from the Immunohaematology and Transfusion Service, S. Orsola-Malpighi University Hospital, Bologna (authorisation 0070755/1980/2014). Availability was granted under conditions complying with Italian privacy law. Neither specific ethics committee approval nor written consent from donors was required for this research project.

### 2.5. Cytotoxicity

The effects of tested compounds on cell viability were monitored by the Cell Counting Kit 8 (WST-8/CCK8) assay (Dojindo Molecular Technologies, Microtech, Italy), as described [20]. DMSO at 10% was used as a cytotoxicity positive control. The assay is based on a production of a formazan dye in response to cellular metabolic activity, measured as absorbance (OD) values. Replicate net OD values were normalised with respect to the control samples and expressed as mean percentage values for cell viability.

### 2.6. Infection

B19V was obtained from a cloned synthetic genome, first transfected into UT7/EpoS1 cells, then propagated by serial passage in EPCs, as described [31]. For infection, EPCs were incubated at a density of 10^7^ cell/mL, in the presence of B19V to a multiplicity of infection (moi, expressed as geq/cell) of 10^3^ geq/cell, for 2 h at 37 °C. After removal of inoculum virus, EPCs were incubated at 37 °C in 5% CO_2_ in complete growth medium, at the different concentrations of tested compounds, at an initial density of 10^6^ cells/mL.

### 2.7. Molecular Analysis

Equal amounts of cell cultures, corresponding to 1.5 × 10^5^ cells, were collected as appropriate at 2 or 48 h post-infection (hpi) and processed by using the Maxwell Viral Total Nucleic Acid kit on a Maxwell MDx platform (Promega), to obtain a total nucleic acid fraction in elution volumes of 150 μL. The quantitative evaluation of target nucleic acids was carried out by qPCR assays in a Rotor-Q system (Qiagen, Hilden, Germany). For the analysis of B19V DNA, aliquots of the eluted nucleic acids (corresponding to ~500 cells) were directly amplified in a qPCR assay (Maxima SYBR Green qPCR Master Mix, Thermo Scientific, Life Technologies, Monza, Italy). For the analysis of B19V RNA, parallel aliquots were first treated with the Turbo DNAfree reagent (Ambion, Life Technologies) before amplification in a qRT-PCR assay (Express One-step SYBR GreenER Kit, Invitrogen, Life Technologies). Standard cycling programs were used, followed by a melting curve analysis to define the $T_{m}$ of amplified products. The primer pair R2210–R2355, located in the central exon of B19V genome, was used to amplify both viral DNA and total RNA, and a target sequence in the region of genomic DNA coding for 5.8S rRNA (rDNA) was amplified in parallel reactions for normalisation [5,31].

## 3. Results

### 3.1. Sequence, Symmetry, and Higher-Order Structures in B19V Genome

The B19V reference sequence used for bioinformatic analysis is a derived consensus sequence, resulting from the alignment of a selected, non-redundant set of complete genomic sequences, referred to as B19V EC [GenBank KY940273]. Such a sequence provides the basis to a synthetic genetic system for B19V, able to yield virus with full replicative competence used for subsequent experiments [31]. The whole genome is 5596 nts long, and its arrangement presents two levels of symmetry. On a genomic scale, a unique internal region, 4830 nts, containing all the coding sequences, is flanked by inverted terminal regions, each 383 nts, serving as replicative origins. Within the terminal regions, the distal 365 nts are disposed as a palindromic sequence around a central site of dyad symmetry. The palindrome is imperfect, presenting a few base mismatches leading to two different sequences, one the inverse complement of the other and usually referred to as “flip-flop”, which can combine independently at each end, thus producing four different sequence isomers.

**Figure 1:**
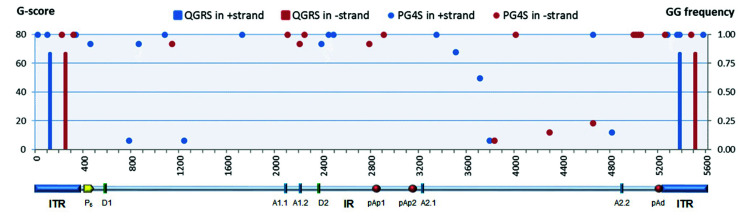
Parvovirus B19 (B19V) genome symmetry and potential quadruplex sequences (PQSs). On a genomic scale, the overall symmetry arrangement includes the two terminal regions flanking the internal unique region. The map of B19V genome shows the two inverted terminal regions (ITR) and the internal region (IR) with the distribution of cis-acting functional sites (P6, promoter; pAp1, pAp2, proximal cleavage-polyadenylation sites; pAd, distal cleavage-polyadenylation site; D1, D2, splice donor sites; A1.1, A1.2, A2.1, A2.2, splice acceptor sites). Superimposed on this arrangement, the B19V genome presents signatures of higher-order structures such as PQSs. Dots (PG4S): GG-PQS in + and − strands, genomic distribution and frequency as reported in the G4-virus database (GG-frequency plotted on right *y*-axis) [28]. Bars (QGRS (Quadruplex forming G-Rich Sequences)): PQSs in + and − strands, genomic distribution and relevance score for PQSs uniquely identified by the QGRS mapper (G-score score plotted on left *y*-axis) [30].

**Figure 2:**
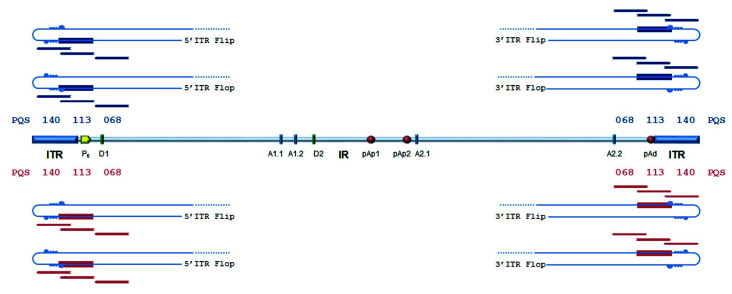
B19V genome symmetry and PQSs. ITRs are shown in the hairpin configuration for the positive and negative strands in the different “flip-flop” isomers. The potential G4 structures predicted by QGRS Mapper are located within the terminal sequences (shown as boxes), 5′ to the dyad symmetry, either on the plus strand (blue) or minus strand (red). PQSs partially overlap with the asymmetries leading to the flip/flop isomers (bubbles). Oligonucleotides used in circular dichroism (CD) experiments (PQSs, Table 1) are shown in context (blue/red stripes). See also Supplementary Figure S1.

Superimposed on these symmetries, the B19V genome presents signatures of higher-order structures such as PQSs (Figure 1). The prediction on the presence and distribution of PQSs in B19V genome reported in the G4-virus database was compared to predictions obtained by the QGRS mapper program. Predictions were only partially concordant. The G4-virus database reports a list of all PQSs identified in the genome, their position on positive and negative strands, their degree of conservation among isolates included in the dataset expressed as frequency, and the statistical significance of their abundance [28]. For B19V, on a dataset of 13 sequences, only the presence of dinucleotide PQS, and not of tri- or tetra-nucleotide PQS, was considered statistically significant over a random distribution. A disperse dinucleotide (GG) PQS distribution was reported, including 22 GG-PQSs in the plus strand and 18 GG-PQSs in the in minus strand, at frequencies in the range 0.08–1.00. On the other hand, the QGRS mapper [30] uniquely identified, at a relevant score (G-score > 60), two sequence stretches with features of a PQS (G_3_N_13_G_3_N_8_G_3_N_11_G_3_) located within the terminal regions, which are characteristically GC-rich. In particular, these PQSs are located on either plus or minus strand, in close 5′ proximity to the axis of dyad symmetry, partially overlapping with the sequence asymmetries and, thus, in different relative positions with respect to “flip” and “flop” isomers (Figure 2).

The palindromic sequences in the terminal regions allow intra-strand base pairing, leading to a hairpin configuration, as well as inter-strand base pairing leading to an extended configuration. Hairpins can provide priming for second-strand synthesis, whereas strands in the extended configuration need to separate and fold back into hairpin structures for reinitiating replication. Predictions locate PQSs within a functional replicative origin, so that the strand unwinding and folding mechanisms occurring during genome replication can offer the opportunity for DNA strands to assume a G4 structure. This, in turn, may play a role in the regulation of viral genome replication or expression. The following experiments analysed the actual propensity of predicted PQSs to assume a G4 configuration, and any potential relevant role of these in the viral lifecycle.

### 3.2. PQSs in B19V DNA: CD Analysis

The propensity of the PQSs identified by the bioinformatic analysis to form G4 structures, which can occur in one of 26 folding arrangements [34], was first investigated by CD spectroscopic analysis on synthetic oligonucleotides of the corresponding sequence (Table 1). CD spectroscopy is routinely employed to investigate the secondary structure of nucleic acids, thanks to its sensitivity to chirality [35], and in this framework it can be used to evaluate the presence and geometry of G4 structures. Each geometry is characterised by different angles for the glycosidic bonds of guanosines and a different topology for the loops linking the stacked tetrads of the G4 stem, defining the coupling among the guanine chromophores of the bases and giving rise to peculiar CD signatures that can be used as an indicator for the presence of G4 structures [36]. Further, CD melting curve analysis [32,33] can yield information on thermal stability and binding of small molecules, in this case G4 ligands such as BRACO-19, a 3,6,9-trisubstitued acridine derivative designed to bind and stabilise quadruplex DNA structures.

**Figure 3:**
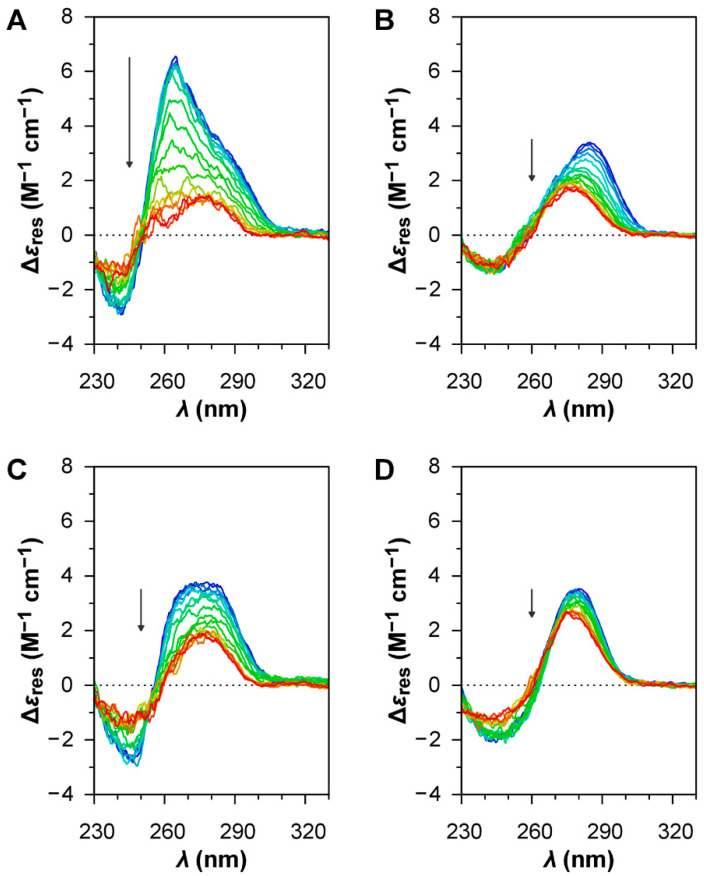
CD spectra of the oligonucleotides under investigation (2 μM) during the heating ramps of the melting assays. (**A**) HIV LTR-II; (**B**) PQS 113; (**C**) PQS 140; (**D**) PQS 068. The arrows indicate the evolution along the heating ramp from 15 to 95 °C.

**Figure 4:**
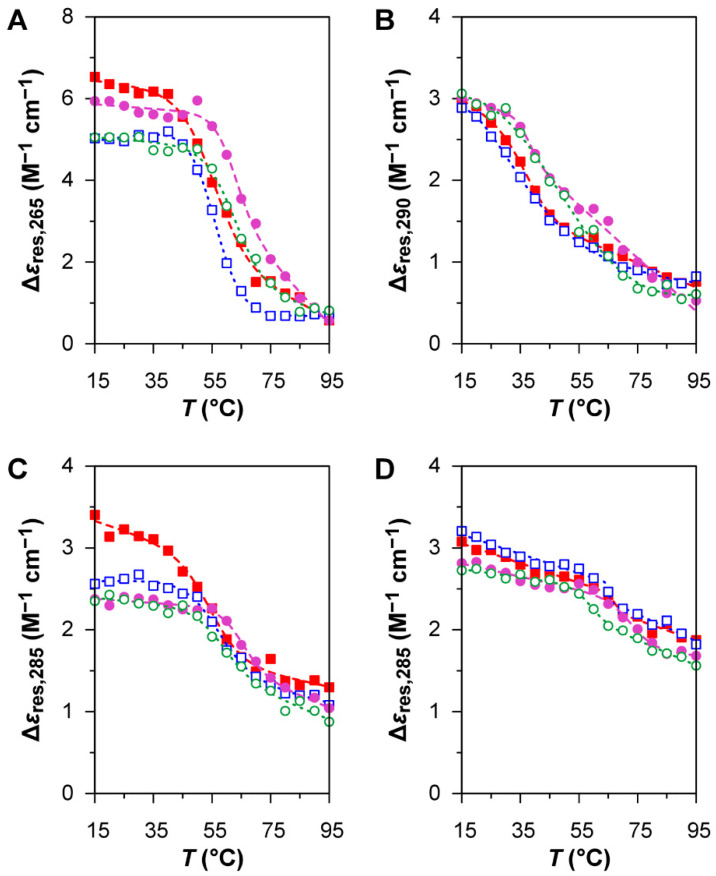
CD melting curves for the oligonucleotides under investigation (2 μM), both in the absence or in the presence of BRACO-19 (10 μM). (**A**) HIV LTR-II; (**B**) PQS 113; (**C**) PQS 140; (**D**) PQS 068. Filled squares: heating ramps (15 to 95 °C) in the absence of BRACO-19. Empty squares: cooling ramps (95 to 15 °C) in the absence of BRACO-19. Filled circles: heating ramps (15 to 95 °C) in the presence of BRACO-19. Empty circles: cooling ramps (95 to 15 °C) in the presence of BRACO-19. BRACO-19—(*N*,*N*′-(9-{[4-(dimethylamino)phenyl]amino}acridine-3,6-diyl)bis(3-pyrrolidin-1-ylpropanamide).

The oligonucleotide HIV LTR-II (Table 1) was chosen as a positive control for the formation of G4 structures [37]. The CD spectrum of this oligonucleotide at low temperature (Figure 3A) can be interpreted as the overlap between the contribution of a parallel G4 structure, which gives a strong positive band centred at around 265 nm [36], and the profile of a GC-rich (76%) ssDNA in B-form, which gives a positive band at around 280 nm and a negative band at around 245 nm [35]. The CD melting curves of the oligonucleotide at 265 nm (Figure 4A), both in the absence and in the presence of BRACO-19, show a clear decrease in intensity for the positive band at 265 nm during the heating ramp, indicative of the disruption of the G4 structure upon thermal denaturation, and a fully reversible profile upon renaturation due to the reorganisation of the G4 structure during the cooling ramp. As expected for a G4 ligand, BRACO-19 stabilises the G4 structure of HIV LTR-II, as the $T_{m}$ of the melting curves is shifted towards higher values (~+7 °C; Table 2), although the degree of stabilisation was found to be smaller than previously reported in the literature [37]. All these observations confirm the presence of a G4 structure in HIV LTR-II.

For B19V, three different oligonucleotides were investigated. Oligo PQS 113 has a sequence matching the most probable PQS in the B19 genome, showing the highest G-score. Oligo PQS 140 has a sequence of corresponding length located in 5′ proximity to the dyad symmetry, upstream and partially overlapping with PQS 113, showing a low G-score. Oligo PQS 068 is also a sequence of corresponding length, located downstream to PQS 113 and showing a null G-score. The oligonucleotides PQS 113, PQS 140, and PQS 068 all display the CD profiles of ssDNA in B-form with no clear contribution from G4 structures (Figure 3B–D); CD signatures peculiar to G4 structures were not observed, while the differences in the CD profiles are most probably due to different primary structures [35].

The CD melting profiles of PQS 113 at 290 nm in the absence of BRACO-19 (Figure 4B) show a broad conformational transition at low temperature after both denaturation and renaturation, revealing a high degree of instability in solution; the large uncertainty of the $T_{m}$ value determined on the cooling ramp (Table 2) is a result of such instability. The CD melting profiles of PQS 140 and PQS 068 in the absence of BRACO-19 (Figure 4C,D) both display narrower, reversible thermal transitions; in both cases, the temperature-dependent variation in CD response at 285 nm has a smaller magnitude than that of HIV LTR-II. The CD melting curves of the oligonucleotides in the presence of BRACO-19 provide an indication of binding, although the underlying mechanisms of these binding interactions appear to be quite different from those observed with HIV LTR-II. Once again, the behaviour of PQS 113 (Figure 4B) is more complex: the trend of the melting curves suggests the possibility of a two-state conformational transition, which is not accurately described by the non-linear regression model used to analyse the melting profiles of G4 structures. On the other hand, the melting curves of PQS 140 and PSQ 068 (Figure 4C,D) are not reversible, since the $T_{m}$ value of the heating ramp is higher than that of the cooling ramp (Table 2). This phenomenon of hysteresis may be explained by a slower kinetics of denaturation and renaturation due to the presence of BRACO-19. Overall, BRACO-19 appears to interact with all the oligonucleotides under investigation, although the mechanism of binding is not consistent with the stabilisation of eventual G4 structures.

### 3.3. PQSs in B19V DNA: Biological Analysis

To extend the results of CD studies and investigate a possible role of putative G4 structures in B19V DNA, we tested the biological effects on the virus–cell system of two reported G4 ligands, BRACO-19 and pyridostatin (PDS) [29]. BRACO-19 has been shown to inhibit telomerase activity, to possess antitumour activity and antiviral activity on different viruses in vitro, including HIV-1. PDS is a very selective G4 DNA-binding small molecule designed to form a complex with and stabilise G4 structures. It has been shown to strongly stabilise telomeric G4, triggering a DNA-damage response at telomeres. As an antiviral agent, PDS has been used to study the role of G4 in Epstein Barr Virus (EBV). As a model cell system, we used primary EPCs, which constitute a heterogeneous cellular population mimicking the natural target cells in in vivo infection and that present full permissiveness to viral replication at the appropriate differentiation stage [5,31]. Effects on cell viability and any possible activity on B19V were assessed in a time course of infection, by evaluating any dose-dependent effects of BRACO-19 and PDS.

**Figure 5:**
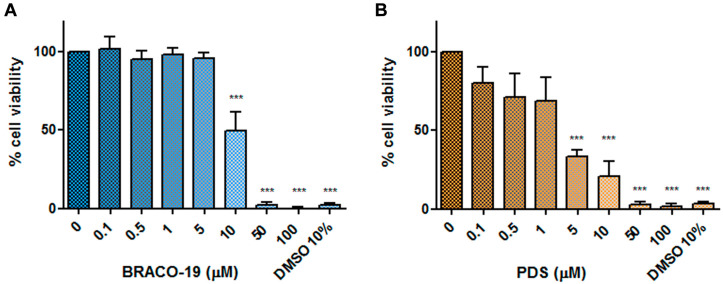
Percentage of viability of erythroid progenitor cells (EPCs) cultured for 48 h in presence of different concentrations of BRACO-19 (**A**) and pyridostatin (PDS) (**B**). DMSO at 10% was used as a cytotoxicity positive control. Values are expressed as mean percentage compared to the control with medium only. Data were collected from triplicate wells in two different experiments. Statistical analysis was performed by one-way ANOVA (analysis of variance) followed by Dunnett’s multiple comparison test. *** *p* value < 0.001.

Effects on cell viability. EPCs were cultured for 48 h at 37 °C in medium containing different concentrations of each compound (0.1–100 μM range), then cell viability was assessed by a WST-8-based colorimetric assay. Results are reported in Figure 5 and are expressed as percentage viability with respect to control cells incubated without compounds. A reduction in cell viability below 50% of control was observed starting from 10 and 5 μM for BRACO-19 and PDS, respectively. At higher concentrations, 50 and 100 μM, the metabolic activity of cells was totally inhibited.

By expressing a dose-dependent relationship between compound concentration and percentage cell viability, non-linear regression curves allowed determining 50% cytotoxic concentration (CC50) values: 9.99 μM for BRACO-19 (95% confidence interval: 8.9–11.53 μM; *R*^2^ = 0.95); 4.01 μM for PDS (95% confidence interval: 0.47–15.52 μM; *R*^2^ = 0.84).

**Figure 6:**
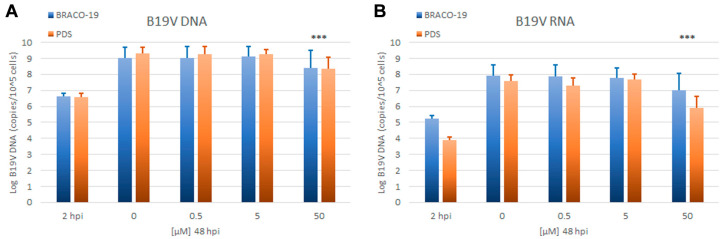
Amount of B19V DNA (**A**) and RNA (**B**) at 2 hpi, before addition of tested compounds, and at 48 hpi in infected cells cultured in the presence of BRACO-19 and PDS at the indicated concentrations (Log copies/10^5^ cells). Data were collected from triplicate qPCR and RT-qPCR reactions in duplicate experiments. Statistical analysis was performed by one-way ANOVA (analysis of variance) followed by Dunnett’s multiple comparison test among 48 hpi samples. *** *p* value < 0.001.

Antiviral activity against B19V: The effects of BRACO-19 and PDS on B19V were evaluated by quantitative determination of viral replication and expression in a time course of infection. EPCs were infected with B19V at the multiplicity of infection of 10^3^ geq/cell and cultured either in the absence or in the presence of each compound, at 0.5, 5, and 50 μM. Cells were collected at 2 and 48 h post-infection (hpi). The extent of viral replication and expression was assessed by qPCR and RT-qPCR evaluation of the number of viral genomes and total transcripts respectively, at 48 hpi compared to that at the baseline at 2 hpi (Figure 6).

Viral DNA increased from 2 to 48 hpi on average 2.5 Log in the control samples, indicating productive viral replication. Inhibition of viral replication, displayed as a significant net reduction in viral DNA from 2 to 48 hpi, was not observed in cells treated with BRACO-19 and PDS. Compared to control samples, a partial inhibition of viral replication was evident only at the highest concentrations, with percentage values of 65% and 85% for BRACO-19 and PDS, respectively. Transcription of the viral genome, as determined by the increase of total viral mRNAs from 2 to 48 hpi, was also unaffected unless at the highest concentrations of BRACO-19 and PDS, with percentage values of 88% and 98%, respectively. The absence of dose-dependent effects on virus and the concomitant and prevalent effects on cell viability suggest that any marginal antiviral activity of BRACO-19 and PDS is likely due to the inhibition of the cellular metabolism rather than to a specific inhibitory activity on the virus, whose replication and expression appears unaffected by these G4 ligands.

## 4. Discussion

B19V is a virus with distinctive features that induces interest, not least in the characterisation of its lifecycle and of virus–host interaction [6]. B19V is a widely circulating human pathogenic virus, although its clinical impact is often underestimated, and the development of specific antiviral tools still suffers from a striking gap. In addition to the propensity to enhanced diagnostic awareness [15], the development of effective antiviral strategies against B19V should be considered a relevant goal in the field [16]. A better understanding of the viral lifecycle and virus–cell interactions are required to identify relevant targets for more efficient and specific antiviral strategies. Unconventional DNA structures, in particular G-quadruplex planar structures disrupting the regular double helix structure of DNA, are increasingly recognised as relevant features for the regulation of critical biological processes. Viruses can include G4-forming sequences in their genomes as part of their interaction network within the cellular environment, and in many instances, these structures can provide targets for small-molecule ligands that can provide an antiviral effect by interfering with the normal viral regulation pathways [27,28,29].

The comprehensive survey in the G4-virus database provides a framework overview of PQS elements in viral genomes, aiming, in its statement, at expediting research on G-quadruplex in viruses, and at finding novel therapeutic opportunities. Out these PQSs, the presence and relevance of G4s as functional elements have been validated in some cases [29], or only predicted otherwise, requiring experimental evidence as in the present case for B19V. For B19V genome, the G4-virus database reports a disperse presence of dinucleotide “GG” PQSs that can be considered statistically significant over a random distribution. An independent prediction on the presence of G4 structures can also be obtained by the QGRS mapper, which identifies at a high score two PQSs within the genomic terminal regions. In our work, we sought to validate these predictions on the presence of PQSs, but neither chemical nor biological evidence could lend experimental support to bioinformatics.

In B19V, predicted PQSs are mainly located within the terminal regions (ITRs), which are critically involved in the viral lifecycle under several aspects [6]. First, ITRs serve as origins of replication of the viral genome. A palindromic sequence is required to allow strand fold-back to form hairpin structures, in turn necessary for priming second-strand synthesis. The sequence asymmetries in the palindrome (flip/flop heterogeneity) are also strictly required [31], as they can possibly induce distortions in the hairpin secondary structure or determine the exact placement of sequence motifs recognised by binding moieties. Moreover, the ITRs are populated by binding motifs for the viral NS and several cellular proteins, relevant for both replication and transcription of the viral genome [38,39,40]. Finally, ITRs have the characteristics of CpG islands and are a possible target for epigenetic modifications such as CpG methylation, in turn able to regulate expression of the viral genome [41]. It should be also mentioned that for viruses in the family, the sole indirect evidence of the presence of PQSs forming G4 structures has been presented for Adeno-associated viruses (AAV) ITRs [42].

Our experimental challenge of bioinformatic predictions analysed the actual propensity of PQSs in B19V DNA to assume G4 structures and any possible inhibitory activity of G4 ligands on the viral lifecycle. Results did not lend support to the bioinformatic predictions on the occurrence of G4 structures in B19V genome and did not show any antiviral role for G4 ligands such as BRACO-19 and PDS. The reason for this discrepancy is possibly due to the sequence and symmetry constraints imposed on the sequence of B19V prevailing over the propensity of forming G4 structures, so that preservation of the secondary hairpin structures within ITRs is likely a more stringent functional requirement than the possibility of forming actual G4 structures. The QGRS mapper is reported to predict G4 structures with high accuracy (>0.95) [26], but for B19V DNA the evidence classifies the predicted PQSs as false positives. Within the ITRs, a high overall GC content may introduce a sequence bias and just increase the probability of detecting PQS-like signatures by bioinformatic tools. Actually, predictions reported in the G4-virus database indicate only a moderate statistical significance of the presence of dinucleotide PQSs. The patterns identified by the QGRS mapper program are not stringent, although the G-score obtained for the PQS regions in B19V ITRs matches that of a validated G4 structure such as HIV LTR-II. Overall, the formation and/or any relevant biological role of unconventional DNA structures such as G4 are unlikely in B19V ITRs, as determined by both CD and in vitro biological studies. On the contrary, it can be hypothesised that the formation of unconventional structures would possibly interfere with many processes crucial to viral replication without conferring any discernible selective advantage. Based on the present data, the development of antiviral strategies directed at perturbing the replicative origins in B19V DNA ITRs cannot include G4 structures as specific targets or G4 ligands as antiviral agents. In this respect, the characteristic combination of hairpin structures and sequence asymmetries appears to be a more relevant feature.

## 5. Conclusions

As a concluding remark, our work highlights how the enormous potential for structural or functional predictions provided by bioinformatic tools must be used with caution and results subjected to critical scrutiny. Computational methods tend to be assertive and additive, especially when aiming at the construction of comprehensive databases based on pattern search algorithms, as in the present case. Experimental validation/falsification of such predictions is required for a correct understanding of the biological systems as well as for the assessment of the computational algorithms’ reliability. As in the present case, a negative experimental evidence is constructive both to avoid misconceptions and to provide a benchmark to evaluate the performance of computational methods.

## Figures and Tables

**Table 1:** Oligonucleotides used for the CD analysis on PQSs in the DNA of B19V.

Oligo	Nts	Sequence	Molecular Weight (Da)	G-Score
HIV LTR-II	33	GGGGACTTTCCAGGGAGGCGTGGCCTGGGCGGG	10,349	68
PQS 140	44	GGGCCAGCTTGCTTGGGGTTGCCTTGACACTAAGACAAGCGGCG	13,630	14
PQS 113	45	GGGACTTCCGGAATTAGGGTTGGCTCTGGGCCAGCTTGCTTGGGG	14,012	67
PQS 068	45	TCATTTCCTGTGACGTCATTTCCTGTGACGTCACTTCCGGTGGGC	13,752	-

**Table 2:** Mid-transition temperatures ($T_{m}$, in °C) for the oligonucleotides under investigation, both in the absence or in the presence of BRACO-19 (10 μM), as determined by CD melting assays.

	*λ*_Δmax_ (nm)	Isolated Oligonucleotide (2 μM)		Oligonucleotide (2 μM) + BRACO-19 (10 μM)	
		15 °C → 95 °C	95 °C → 15 °C	15 °C → 95 °C	95 °C → 15 °C
HIV LTR-II	265	55.0 ± 1.4	55.9 ± 0.4	61.8 ± 1.5	62.4 ± 1.6
PQS 113	290	37.4 ± 2.4	0.3 ± 81.5	37.3 ± 2.5	45.3 ± 16.7
PQS 140	285	54.6 ± 2.3	56.2 ± 1.8	64.7 ± 1.7	57.8 ± 2.8
PQS 068	285	67.3 ± 2.8	65.0 ± 6.6	73.3 ± 3.3	58.1 ± 1.4

BRACO-19—(*N*,*N*′-(9-{[4-(dimethylamino)phenyl]amino}acridine-3,6-diyl)bis(3-pyrrolidin-1-ylpropanamide).

## Supplementary Materials

The following are available online at https://www.mdpi.com/1999-4915/12/9/935/s1. Figure S1: Inverted Terminal Regions (ITR) in B19V genome.
