# Supplementary material for: No G-Quadruplex Structures in the DNA of Parvovirus B19: Experimental Evidence versus Bioinformatic Predictions
Source: Viruses. 2020 Aug 25;12(9):935. doi: 10.3390/v12090935 (PMC7552014; doi:10.3390/v12090935)
Supplement: Supplementary file 1 [file viruses-12-00935-s001.pdf]

PQS in B19V ITRs: Hairpin configuration, Left End

+strand Flip

```
CGCCGCTTG-TCTTAGTGTCAAGGCAACCCCAAGCAAGCTGGCCCAGAGCCAACCCTAATTCCGGAAGTCCCGCCCACCGGAAGTGACGTCACAGGAAATGACGTCACAGGAAATGACGTAATTGTCCGCCATCTTGTACCGGAAGTCCCGCCTACCGGCGGCGACCGGCGGCATCTGATTTGGTGTCTTCTTTTAAATTTT -  3’
||||||||| |||||||| || | |||||||||||  ||||||||||||||||||||||||||||||||||||||||||||||||||||||||||||||||||||||||||||||||||||||||||||||||||||||||||||||||||||||||||||||||||||||||||||||||
GCGGCGAACTAGAATCACCGTGCAGTTGGGGTTCG--CGACCGGGTCTCGGTTGGGATTAAGGCCTTCAGGGCGGGTGGCCTTCACTGCAGTGTCCTTTACTGCAGTGTCCTTTACTGCATTAACAGGCGGTAGAACATGGCCTTCAGGGCGGATGGCCGCCGCTGGCCGCCGTAGACTAAACC -                  5’

<<<<<<<<<<<<<<<<<<<<<<<<<<<<<<<<<<<  <<<<<<<<                                                                                                                                           PQS140
                           <<<<<<<<  <<<<<<<<<<<<<<<<<<<<<<<<<<<<<<<<<<<                                                                                                                    PQS113
                                                                        <<<<<<<<<<<<<<<<<<<<<<<<<<<<<<<<<<<<<<<<<<<<<                                                                       PQS068
```

+strand Flop

```
CGCCGCTTGATCTTAGTGGCACGTCAACCCCAAGC--GCTGGCCCAGAGCCAACCCTAATTCCGGAAGTCCCGCCCACCGGAAGTGACGTCACAGGAAATGACGTCACAGGAAATGACGTAATTGTCCGCCATCTTGTACCGGAAGTCCCGCCTACCGGCGGCGACCGGCGGCATCTGATTTGGTGTCTTCTTTTAAATTTT -  3’
||||||||| |||||||| || | |||||||||||  ||||||||||||||||||||||||||||||||||||||||||||||||||||||||||||||||||||||||||||||||||||||||||||||||||||||||||||||||||||||||||||||||||||||||||||||||
GCGGCGAAC-AGAATCACAGTTCCGTTGGGGTTCGTTCGACCGGGTCTCGGTTGGGATTAAGGCCTTCAGGGCGGGTGGCCTTCACTGCAGTGTCCTTTACTGCAGTGTCCTTTACTGCATTAACAGGCGGTAGAACATGGCCTTCAGGGCGGATGGCCGCCGCTGGCCGCCGTAGACTAAACC -                  5’

<<<<<<<<<<<<<<<<<<<<<<<<<<<<<<<<<<<  <<<<<<<<                                                                                                                                           PQS140
                           <<<<<<<<  <<<<<<<<<<<<<<<<<<<<<<<<<<<<<<<<<<<                                                                                                                    PQS113
                                                                        <<<<<<<<<<<<<<<<<<<<<<<<<<<<<<<<<<<<<<<<<<<<<                                                                       PQS068
```

-strand Flop

```
CGCCGCTTGATCTTAGTGGCACGTCAACCCCAAGC--GCTGGCCCAGAGCCAACCCTAATTCCGGAAGTCCCGCCCACCGGAAGTGACGTCACAGGAAATGACGTCACAGGAAATGACGTAATTGTCCGCCATCTTGTACCGGAAGTCCCGCCTACCGGCGGCGACCGGCGGCATCTGATTTGG -                  3’
||||||||| |||||||| || | |||||||||||  ||||||||||||||||||||||||||||||||||||||||||||||||||||||||||||||||||||||||||||||||||||||||||||||||||||||||||||||||||||||||||||||||||||||||||||||||
GCGGCGAAC-AGAATCACAGTTCCGTTGGGGTTCGTTCGACCGGGTCTCGGTTGGGATTAAGGCCTTCAGGGCGGGTGGCCTTCACTGCAGTGTCCTTTACTGCAGTGTCCTTTACTGCATTAACAGGCGGTAGAACATGGCCTTCAGGGCGGATGGCCGCCGCTGGCCGCCGTAGACTAAACCACAGAAGAAAATTTAAAA -  5’

<<<<<<<<<<<<<<<<<<<<<<<<<<<<<<<<<<<  <<<<<<<<                                                                                                                                           PQS140
                           <<<<<<<<  <<<<<<<<<<<<<<<<<<<<<<<<<<<<<<<<<<<                                                                                                                    PQS113
                                                                        <<<<<<<<<<<<<<<<<<<<<<<<<<<<<<<<<<<<<<<<<<<<<                                                                       PQS068
```

-strand Flip

```
CGCCGCTTG-TCTTAGTGTCAAGGCAACCCCAAGCAAGCTGGCCCAGAGCCAACCCTAATTCCGGAAGTCCCGCCCACCGGAAGTGACGTCACAGGAAATGACGTCACAGGAAATGACGTAATTGTCCGCCATCTTGTACCGGAAGTCCCGCCTACCGGCGGCGACCGGCGGCATCTGATTTGG -                  3’
||||||||| |||||||| || | |||||||||||  ||||||||||||||||||||||||||||||||||||||||||||||||||||||||||||||||||||||||||||||||||||||||||||||||||||||||||||||||||||||||||||||||||||||||||||||||
GCGGCGAACTAGAATCACCGTGCAGTTGGGGTTCG--CGACCGGGTCTCGGTTGGGATTAAGGCCTTCAGGGCGGGTGGCCTTCACTGCAGTGTCCTTTACTGCAGTGTCCTTTACTGCATTAACAGGCGGTAGAACATGGCCTTCAGGGCGGATGGCCGCCGCTGGCCGCCGTAGACTAAACCACAGAAGAAAATTTAAAA -  5’

<<<<<<<<<<<<<<<<<<<<<<<<<<<<<<<<<<<  <<<<<<<<                                                                                                                                           PQS140
                           <<<<<<<<  <<<<<<<<<<<<<<<<<<<<<<<<<<<<<<<<<<<                                                                                                                    PQS113
                                                                        <<<<<<<<<<<<<<<<<<<<<<<<<<<<<<<<<<<<<<<<<<<<<                                                                       PQS068
```

PQS in B19V ITRs: Hairpin configuration, Right End

+strand Flip

```
PQS068                                                                                     >>>>>>>>>>>>>>>>>>>>>>>>>>>>>>>>>>>>>>>>>>>>
PQS113                                                                                                                                 >>>>>>>>>>>>>>>>>>>>>>>>>>>>>>>>>>>--
>>>>>>>>
PQS140                                                                                                                                                            >>>>>>>>-->>>>>>>>>>>>>>>>>>>>>>>>>>>>>>>>>>>

5’ – AAAATTTAAAAGAAGACACCAAATCAGATGCCGCCGGTCGCCGCCGGTAGGCGGGACTTCCGGTACAAGATGGCGGACAATTACGTCATTTCCTGTGACGTCATTTCCTGTGACGTCACTTCCGGTGGGCGGGACTTCCGGAATTAGGGTTGGCTCTGGGCCAGCTTGCTTGGGGTTGCCTTGACACTAAGA-CAAGCGGCG
                       ||||||||||||||||||||||||||||||||||||||||||||||||||||||||||||||||||||||||||||||||||||||||||||||||||||||||||||||||||||||||||||||||||||||||||||||||||  ||||||||||| | || |||||||| |||||||||
3’                   – GGTTTAGTCTACGGCGGCCAGCGGCGGCCATCCGCCCTGAAGGCCATGTTCTACCGCCTGTTAATGCAGTAAAGGACACTGCAGTAAAGGACACTGCAGTGAAGGCCACCCGCCCTGAAGGCCTTAATCCCAACCGAGACCCGGTCG--CGAACCCCAACTGCACGGTGATTCTAGTTCGCCGC
```

+strand Flop

```
PQS068                                                                                     >>>>>>>>>>>>>>>>>>>>>>>>>>>>>>>>>>>>>>>>>>>>
PQS113                                                                                                                                 >>>>>>>>>>>>>>>>>>>>>>>>>>>>>>>>>>>-->>>>>>>>
PQS140                                                                                                                                                            >>>>>>>>-->>>>>>>>>>>>>>>>>>>>>>>>>>>>>>>>>>>

5’ – AAAATTTAAAAGAAGACACCAAATCAGATGCCGCCGGTCGCCGCCGGTAGGCGGGACTTCCGGTACAAGATGGCGGACAATTACGTCATTTCCTGTGACGTCATTTCCTGTGACGTCACTTCCGGTGGGCGGGACTTCCGGAATTAGGGTTGGCTCTGGGCCAGC--GCTTGGGGTTGACGTGCCACTAAGATCAAGCGGCG
                       ||||||||||||||||||||||||||||||||||||||||||||||||||||||||||||||||||||||||||||||||||||||||||||||||||||||||||||||||||||||||||||||||||||||||||||||||||  ||||||||||| | || |||||||| |||||||||
3’                   – GGTTTAGTCTACGGCGGCCAGCGGCGGCCATCCGCCCTGAAGGCCATGTTCTACCGCCTGTTAATGCAGTAAAGGACACTGCAGTAAAGGACACTGCAGTGAAGGCCACCCGCCCTGAAGGCCTTAATCCCAACCGAGACCCGGTCGAACGAACCCCAACGGAACTGTGATTCT-GTTCGCCGC
```

-strand Flop

```
PQS068                                                                                     >>>>>>>>>>>>>>>>>>>>>>>>>>>>>>>>>>>>>>>>>>>>
PQS113                                                                                                                                 >>>>>>>>>>>>>>>>>>>>>>>>>>>>>>>>>>>-->>>>>>>>
PQS140                                                                                                                                                            >>>>>>>>-->>>>>>>>>>>>>>>>>>>>>>>>>>>>>>>>>>>

5’                   – CCAAATCAGATGCCGCCGGTCGCCGCCGGTAGGCGGGACTTCCGGTACAAGATGGCGGACAATTACGTCATTTCCTGTGACGTCATTTCCTGTGACGTCACTTCCGGTGGGCGGGACTTCCGGAATTAGGGTTGGCTCTGGGCCAGC--GCTTGGGGTTGACGTGCCACTAAGATCAAGCGGCG
                       ||||||||||||||||||||||||||||||||||||||||||||||||||||||||||||||||||||||||||||||||||||||||||||||||||||||||||||||||||||||||||||||||||||||||||||||||||  ||||||||||| | || |||||||| |||||||||
3’ – TTTTAAATTTTCTTCTGTGGTTTAGTCTACGGCGGCCAGCGGCGGCCATCCGCCCTGAAGGCCATGTTCTACCGCCTGTTAATGCAGTAAAGGACACTGCAGTAAAGGACACTGCAGTGAAGGCCACCCGCCCTGAAGGCCTTAATCCCAACCGAGACCCGGTCGAACGAACCCCAACGGAACTGTGATTCT-GTTCGCCGC
```

-strand Flip

```
PQS068                                                                                     >>>>>>>>>>>>>>>>>>>>>>>>>>>>>>>>>>>>>>>>>>>>
PQS113                                                                                                                                 >>>>>>>>>>>>>>>>>>>>>>>>>>>>>>>>>>>-->>>>>>>>
PQS140                                                                                                                                                            >>>>>>>>-->>>>>>>>>>>>>>>>>>>>>>>>>>>>>>>>>>>

5’                   – CCAAATCAGATGCCGCCGGTCGCCGCCGGTAGGCGGGACTTCCGGTACAAGATGGCGGACAATTACGTCATTTCCTGTGACGTCATTTCCTGTGACGTCACTTCCGGTGGGCGGGACTTCCGGAATTAGGGTTGGCTCTGGGCCAGCTTGCTTGGGGTTGCCTTGACACTAAGA-CAAGCGGCG
                       ||||||||||||||||||||||||||||||||||||||||||||||||||||||||||||||||||||||||||||||||||||||||||||||||||||||||||||||||||||||||||||||||||||||||||||||||||  ||||||||||| | || |||||||| |||||||||
3’ – TTTTAAATTTTCTTCTGTGGTTTAGTCTACGGCGGCCAGCGGCGGCCATCCGCCCTGAAGGCCATGTTCTACCGCCTGTTAATGCAGTAAAGGACACTGCAGTAAAGGACACTGCAGTGAAGGCCACCCGCCCTGAAGGCCTTAATCCCAACCGAGACCCGGTCG--CGAACCCCAACTGCACGGTGATTCTAGTTCGCCGC
```

LEGEND

Inverted Terminal Regions (ITR) in B19V genome. The left and right ends are shown in the hairpin configuration for positive and negative sense strands, in the ‘flip’ or flop’ configuration.

In yellow, the regions of imperfect base pairing giving rise to the ‘flip’ and flop’ configurations. Underlined, the PG4S regions predicted by QGRS mapper, in bold the G residues involved.

Gallons indicate the position of the three oligonucleotides used for CD analysis (PQS140, PQS113, PQS068). The PG4S and corresponding oligonucleotides match only one of the strands in the hairpin stem.
